# On the Hunt for Vascular Endothelial Stem Cells

**DOI:** 10.1371/journal.pbio.1001408

**Published:** 2012-10-16

**Authors:** Caitlin Sedwick

**Affiliations:** Freelance Science Writer, San Diego, California, United States of America

**Figure pbio-1001408-g001:**
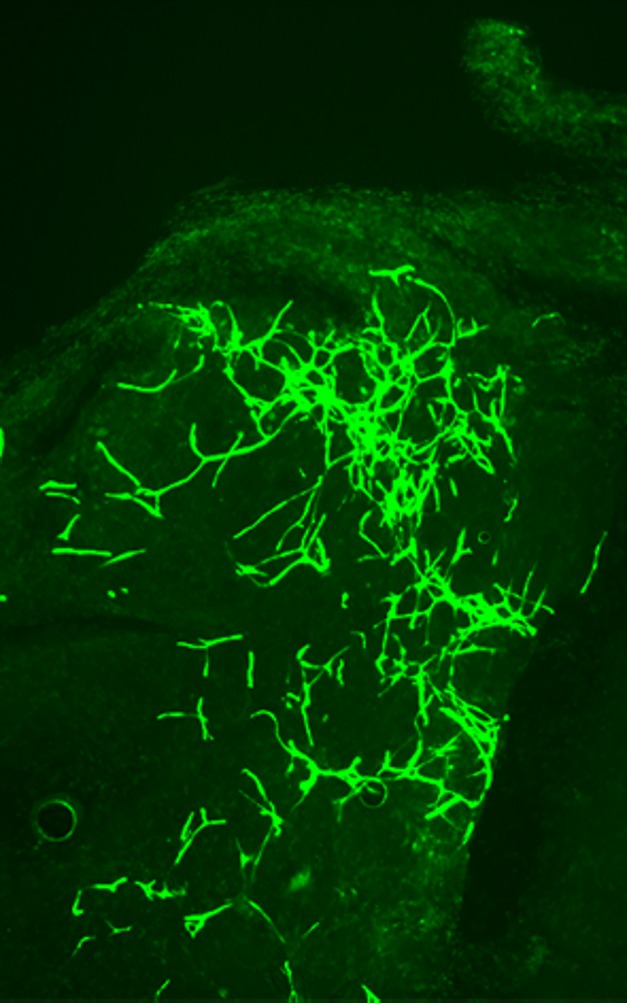
A single adult endothelial stem cell with the c-kit marker can generate functional blood vessels. The illustration shows GFP+ blood vessels generated subcutaneously in a mouse by the transplanted descendants of a single genetically GFP-tagged endothelial stem cell. (Image: Fang, Wei, and Salven).

[Fig pbio-1001408-g001]In the inner blastocyst of early embryos are found pluripotent stem cells, which have the potential to turn into every type of cell in the adult animal. Later in development, stem cells still exist, but they produce a more restricted range of progeny cell types. Tissue-specific stem cells arise that contribute primarily to individual tissues such as skin, muscle, or blood. These cells are essential both during development, when they deliver differentiated progeny to fulfill the demands of tissue growth, and in adulthood, when they swing into action to replace cells lost to age or to injury.

It's likely that every adult tissue possesses a population of tissue-specific stem cells, but because they tend to be quite rare, these adult stem cells have not yet been identified for all tissues. For example, much effort has been devoted toward isolating and characterizing the adult stem cells that give rise to the vascular endothelium (the inner lining of blood vessels). Knowing how to identify these cells could make it possible to manipulate endothelial cell proliferation, which in turn could aid in the development of therapies to promote vascular repair or to prevent blood vessel growth within tumors. Unfortunately, such efforts have met with limited success. But in this issue of *PLOS Biology*, Shentong Fang, Petri Salven, and colleagues have identified a population of cells that have all the characteristics expected of vascular endothelial stem cells (VESC), opening the way for a better understanding of these important cells.

For some time, it was thought that the stem cells that give rise to the vascular endothelium might not actually be residents of that tissue. In fact, it was thought that hematopoietic stem cells, which produce immune cells and red blood cells, could also produce cells that become vascular endothelial cells. In support of this idea, it was shown that some kinds of cells found in blood are highly proliferative and express cell surface proteins typical of endothelial cells. However, later experiments showed that cells derived from fluorescently marked hematopoietic stem cells never appear in the vascular endothelium, suggesting VESC probably reside within the vascular endothelium. But the characteristics of such cells—and what (if any) surface proteins might set them apart from other endothelial cells—remained a mystery.

To identify and isolate VESCs, Fang and colleagues took advantage of the fact that, while the highly differentiated cells that make up the bulk of most tissues can only undergo a limited number of cell divisions, stem cells have the ability to renew themselves. The authors created single-cell suspensions from enzymatically digested lung vasculature that had been carefully cleared of blood cells, and looked for cells with the high proliferative capability required to form colonies in cell culture. These experiments uncovered a set of very rare colony-forming cells. Subsequent analysis established that these cells express common endothelial cell markers and also display the cell surface protein CD117 (c-kit), a marker that's been found on other types of stem cells.

These findings demonstrated that some CD117-positive cells display a defining property of stem cells: the capacity to self-renew. This ability is restricted to CD117-expressing cells; when freshly isolated endothelial cells were sorted into CD117-positive and -negative populations, the only cells that could grow in culture came from the CD117-positive population. But only about one half of one percent of CD117-expressing cells isolated from vascular endothelium have this property, which fits with the idea that these stem cells are rather rare.

The authors showed that CD117-positive cells are not simply an artifact of culture conditions, because they are present in the vascular endothelium of several normal mouse tissues and in the tumor vasculature of both mouse and human cancers. What's more, mice with impaired CD117 expression have deficient vasculature growth. These animals also exhibit slower growth of implanted melanoma tumors due to decreased tumor blood vessel growth. Meanwhile, a single colony of CD117-positive colony-forming cells plucked from culture can give rise to entire blood vessels when seeded into a plug of extracellular matrix material that has been implanted into a mouse host. In agreement with this, Fang et al. showed that these cells can also form blood vessels in solid tumors implanted into a mouse host.

Taken together, the authors' data suggest that CD117-positive colony forming cells possess both the self-renewing capacity and the versatility expected of adult stem cells, and the evidence points to these cells being the long-sought-after VESC. With this information in hand, it should now be possible to determine what other markers set this stem cell population apart, leading to a better understanding of this critical stem cell population and paving the way for therapeutic benefits down the road.

**Fang S, Wei J, Pentinmikko N, Leinonen H, Salven P (2012) Generation of Functional Blood Vessels from a Single c-kit+ Adult Vascular Endothelial Stem Cell. doi:10.1371/journal.pbio.1001407**

